# Improved Development of Somatic Cell Cloned Mouse Embryos by Vitamin C and Latrunculin A

**DOI:** 10.1371/journal.pone.0120033

**Published:** 2015-03-06

**Authors:** Anna Mallol, Josep Santaló, Elena Ibáñez

**Affiliations:** Departament de Biologia Cellular, Fisiologia i Immunologia, Facultat de Biociències, Universitat Autònoma de Barcelona, Bellaterra, Spain; USA, UNITED STATES

## Abstract

Impaired development of embryos produced by somatic cell nuclear transfer (SCNT) is mostly associated with faulty reprogramming of the somatic nucleus to a totipotent state and can be improved by treatment with epigenetic modifiers. Here we report that addition of 100 μM vitamin C (VitC) to embryo culture medium for at least 16 h post-activation significantly increases mouse blastocyst formation and, when combined with the use of latrunculin A (LatA) during micromanipulation and activation procedures, also development to term. In spite of this, no significant effects on pluripotency (OCT4 and NANOG) or nuclear reprogramming markers (H3K14 acetylation, H3K9 methylation and DNA methylation and hydroxymethylation) could be detected. The use of LatA alone significantly improved *in vitro* development, but not full-term development. On the other hand, the simultaneous treatment of cloned embryos with VitC and the histone deacetylase inhibitor psammaplin A (PsA), in combination with the use of LatA, resulted in cloning efficiencies equivalent to those of VitC or PsA treatments alone, and the effects on pluripotency and nuclear reprogramming markers were less evident than when only the PsA treatment was applied. These results suggest that although both epigenetic modifiers improve cloning efficiencies, possibly through different mechanisms, they do not show an additive effect when combined. Improvement of SCNT efficiency is essential for its applications in reproductive and therapeutic cloning, and identification of molecules which increase this efficiency should facilitate studies on the mechanism of nuclear reprogramming and acquisition of totipotency.

## Introduction

Somatic cell nuclear transfer (SCNT), to be successful, requires the transferred somatic nucleus to undergo an orchestrated array of epigenetic modifications leading to changes in the structure and composition of chromatin. This epigenetic reprogramming, driven by factors present in the cytoplasm of the recipient oocyte, allows the acquisition of an undifferentiated embryo-like chromatin status compatible with embryonic gene activation and embryonic development [1, 2]. Despite the birth of cloned animals from a variety of species, the low efficiency of SCNT suggests that epigenetic reprogramming of the somatic nucleus is often incomplete or incorrect, leading to the developmental arrest of the cloned embryos. Indeed, aberrant DNA methylation and histone modification patterns have been frequently reported in SCNT embryos [3–9].

In light of the epigenetic abnormalities found in cloned embryos, a new SCNT protocol was developed to improve reprogramming of the somatic nucleus: the treatment of the donor somatic cells or, more often, the reconstructed oocytes with epigenetic modifiers. So far, the most significant results have been achieved with inhibitors of histone deacetylases (HDACi), such as trichostatin A, scriptaid, valproic acid or oxamflatin [10–17]. These HDACi treatments increase histone acetylation levels, resulting in improved chromatin remodeling and accessibility for replication and transcription factors and, consequently, in significantly higher cloning efficiencies [18]. The effects on nuclear reprogramming and cloning efficiencies of other epigenetic modifiers, such as inhibitors of DNA or histone methyltransferases, is still uncertain [19–22]. Other recent technical improvements to the SCNT protocol, such as the use of the actin polymerization inhibitor latrunculin A (LatA) instead of cytochalasin B (CB) to prevent second polar body extrusion, have also contributed to enhanced cloning efficiencies when combined with HDACi treatments [23–25]. But despite these advancements, the developmental potential of cloned embryos is still suboptimal, hindering the widespread application of SCNT technology.

Vitamin C (VitC), also known as L-ascorbic acid, is a well-known antioxidant that protects the cells against oxidative stress. Many reports have shown its beneficial effects when added to oocyte maturation or embryo culture media [26–31]. Recent studies have shown that VitC has also a positive role in nuclear reprogramming of differentiated cells towards a pluripotent state. In particular, VitC improves the generation of induced pluripotent stem cells (iPSCs) from both mouse and human somatic cells [32] and promotes the conversion of partially reprogrammed mouse iPSCs to a fully reprogrammed state [33]. The effect of VitC on reprogramming seems to be independent of its antioxidant effect and may rather be explained by a combined enhancement of Jumonji-domain-containing histone demethylases (JHDMs) and ten-eleven translocation (TET) methylcytosine dioxygenases activities [34–37], which promote histone and DNA demethylation, respectively, and lead to changes in chromatin structure and gene expression [38, 39]. The positive role of VitC on the *in vitro* development of SCNT porcine embryos and their nuclear reprogramming, in terms of histone acetylation and pluripotency markers expression, has also been proved [29, 31, 40], but its effects on DNA and histone methylation and full-term development of the cloned embryos has yet to be demonstrated. On the other hand, VitC seems not to improve the *in vitro* development of SCNT embryos from other mammalian species, but rather negatively affect it [41, 42].

We have recently reported that the HDACi psammaplin A (PsA), a natural compound isolated from a marine sponge [43], results in enhanced rates of full-term development of cloned mouse embryos [25]. Given that PsA and VitC act on different epigenetic modifying enzymes, and that the cooperation of multiple pathways is needed to coordinate reprogramming of various epigenetic modifications during SCNT [39], we hypothesized that both compounds could act synergistically in improving nuclear reprogramming of SCNT embryos. On the other hand, the antioxidant role of VitC may enhance the HDAC inhibitory activity of PsA, as it has been reported that reduction of PsA after its uptake into cells is essential for its HDACi activity [44].

Thus, the aims of this study were to explore the effect of VitC on mouse SCNT efficiency, and to investigate whether the combination of VitC and PsA treatments has a synergistic effect. To this aim, SCNT mouse embryos were treated with VitC, PsA or the combination of both (VitC-PsA) and *in vitro* development, blastocyst cell numbers, epigenetic modifications (H3K14 acetylation, H3K9 dimethylation and DNA methylation and hydroxymethylation), levels of pluripotency (OCT4 and NANOG) and trophectoderm (CDX2) markers, reduced glutathione content, and full-term development were assessed. Additionally, the effect of LatA on both *in vitro* and *in vivo* development of untreated and treated embryos was investigated.

## Material and Methods

Unless otherwise indicated, all reagents were purchased from Sigma (Madrid, Spain).

### Animals

Mouse care and procedures were conducted according to the protocols approved by the Ethics Committee on Animal and Human Research of the *Universitat Autònoma de Barcelona* and by the *Departament de Medi Ambient i Habitatge* of the *Generalitat de Catalunya* (Permit numbers: 6064 and 5044).

Hybrid B6CBAF1 (C57Bl/6xCBA/J) mice aged 6–12 weeks were used as sperm, oocytes and cumulus cells donors. Outbred CD1 females mated with normal or vasectomised CD1 males were used as foster or surrogate mothers, respectively. All animals were purchased from Charles River (L’Arbresle, France).

### Collection of sperm, oocytes and cumulus cells

Spermatozoa were collected from the cauda epididymes in a drop (500 μl /epididymis) of Hepes-buffered CZB medium (HCZB) [45] covered with oil, and were allowed to disperse during 20 min at 37°C. The sperm suspension was then diluted in vials of fresh HCZB (1:2 dilution), which were directly plunged into liquid nitrogen without cryoprotection. Sperm samples were stored at -80°C for up to one month.

Oocytes and cumulus cells were collected just before use. Females were induced to superovulate by intraperitoneal injection of 5 IU of pregnant mare serum gonadotropin (Intervet, Barcelona, Spain) followed 48 h later by 5 IU of human chorionic gonadotropin (hCG; Farma-Lepori, Barcelona, Spain). Metaphase II oocytes were collected from the oviducts 14–15 h after hCG administration in HCZB, and treated with 300 U/ml hyaluronidase in HCZB at 37°C until dispersion of cumulus cells. Denuded oocytes were then washed and kept in drops of KSOM culture medium (MR-106-D; Millipore, Madrid, Spain) covered with mineral oil at 37°C under 5% CO_2_. Dispersed cumulus cells were removed from hyaluronidase, diluted in HCZB and centrifuged for 5 min at 250 g. The pellet was then resuspended in a small volume of 3% (v/v) polyvinylpyrrolidone (PVP) in HCZB and kept at 4°C until use.

### Intracytoplasmic sperm injection (ICSI)

Sperm samples were thawed at room temperature and 5–10 μl of the concentrated sperm suspension were introduced into a microdrop of 3% PVP. Oocytes were transferred to a microdrop of HCZB and injected with a sperm head, decapitated by the freeze/thaw procedure [46]. Injections were performed using blunt-end mercury-filled pipettes (outer diameter 5–6 μm) attached to a piezo impact drill (Burleigh, Mississauga, Canada) driven by a micromanipulation device (TransferMan NK2; Eppendorf, Hamburg, Germany) installed on an Olympus IX71 microscope (Olympus, Hospitalet del Llobregat, Spain). Injected oocytes were allowed to recover for 15 min and then were extensively washed and cultured in KSOM.

### Somatic cell nuclear transfer (SCNT)

Oocytes were enucleated and injected with a cumulus cell nucleus using blunt-end mercury-filled pipettes (outer diameter 8–10 μm and 5–6 μm, respectively) attached to a piezo impact drill driven by the aforementioned micromanipulation equipment. Micromanipulations were performed in groups of 25–30 oocytes in drops of HCZB containing either 5 μg/ml CB or 5 μM LatA (Santa Cruz Biotechnology, Heidelberg, Germany). Afterwards, the reconstructed oocytes were extensively washed and cultured for 1–3 h in KSOM supplemented or not with the epigenetic modifiers before activation.

Chemical activation of reconstructed oocytes was performed by incubating them for 6 h (37°C, 5% CO_2_) in 10 mM SrCl_2_ in Ca^2+^free-CZB medium supplemented with either 5 μg/ml CB or 5 μM LatA to prevent second polar body extrusion. After activation, oocytes showing at least one pronucleus were extensively washed and cultured in KSOM with or without the epigenetic modifiers.

### Epigenetic modifier treatments of cloned embryos

VitC (L-ascorbic acid, Sigma A5960) was dissolved in tri-distilled water to prepare a 5 mg/ml stock solution, filtered, and stored at 4°C for up to 3 weeks. PsA (Santa Cruz Biotechnology 258049, Heidelberg, Germany) was dissolved in DMSO to prepare a 3 mM stock solution and stored frozen. The final concentrations of both epigenetic modifiers were prepared by dilution of the stock solutions in the culture or activation media, depending on the experimental procedure. Embryos were exposed to 100 μM VitC during 2–3 h after reconstruction and 6 h of activation (8–9 h treatment) or during 6 h of activation and 10 h, 18 h or the whole duration of the posterior culture (16 h, 24 h and 120 h treatments, respectively). Embryos were exposed to 10 μM PsA or the combination of both epigenetic modifiers (VitC-PsA) during 6 h of activation and 10 h of the posterior culture (16 h treatment). At the end of the treatments, embryos were extensively washed in drops of KSOM culture medium.

### Blastocyst differential staining and cell counts

Cloned embryos that reached the blastocyst stage 96 h post-activation in the first set of experiments were used for the differential staining of trophectoderm (TE) and inner cell mass (ICM) cells, as previously described [47]. Samples were examined with an Olympus IX71 epifluorescence microscope fitted with an image capture system (Cell A 2.6, Olympus, Hospitalet del Llobregat, Spain). ImageJ software (Image J 1.45q, Wayne Rasband, National Institutes of Health, Bethesda, USA) was used for cell counting.

### Immunofluorescence staining, image analysis and cell counts

Cloned and ICSI embryos at different developmental stages were fixed and processed for immunofluorescence detection of H3K14 acetylation (H3K14ac), H3K9 dimethylation (H3K9me2), 5-methylcytosine (5meC), OCT4, NANOG and CDX2 using previously described protocols [25]. The immunofluorescence detection of 5-hydroxymethylcytosine (5hmeC) was also performed, by incubating previously fixed, permeabilized, blocked and 4N HCl-treated embryos first with a 1:500 dilution of rabbit polyclonal anti-5hmeC primary antibody (Active motif, La Hulpe, Belgium) overnight at 4°C, and then with 6 μg/mL goat anti-rabbit Alexa Fluor 594 secondary antibody (Molecular probes, Life technologies, Alcobendas, Spain) for 1h at room temperature.

H3K14ac was analyzed in cloned embryos at 10 min and 1 h after nuclear transfer, and in cloned and control ICSI embryos at 16 h, 24 h and 120 h after activation by parthenogenesis or sperm injection, respectively. H3K9me2 and 5meC were detected in cloned and control ICSI embryos at 72 h and 120 h after activation. 5meC was also detected at 6 h and 16 h after activation along with 5hmeC. Finally, NANOG, OCT4 and CDX2 markers were analyzed in cloned and ICSI blastocysts at 120 h after activation. For each marker analyzed, 15–25 embryos were used and were stained simultaneously to reduce experimental variability.

All samples were examined with an Olympus Bx41 (Olympus, Hospitalet del Llobregat, Spain) epifluorescence microscope fitted with specific filters and an image capture and analyzing system (Isis software version 5.4.5, Metasystems, Boston, USA). For each antibody, images were acquired using the same exposure times and settings for all embryos and analyzed with ImageJ software (Image J 1.45q, Wayne Rasband, National Institutes of Health, Bethesda, USA) for cell counts (NANOG and OCT4/CDX2 double staining of blastocysts) and fluorescence quantification. All individual nuclei of embryos at 1-cell, 2-cell and morula stages and 30 nuclei of random regions per blastocyst were outlined for mean fluorescence intensity calculation.

### Quantification of reduced glutathione content in cloned embryos

Non-fluorescent monochlorobimane (MCB) bounds to reduced glutathione (GSH) in a reaction catalyzed by glutathione-s-transferase and forms a fluorescent adduct whose fluorescence intensity gives a measure of GSH content. Non-treated and treated cloned embryos (18–21 embryos per group) at 16 h post-activation were incubated with 50 μM MCB in KSOM for 40 min at 37°C. Then, embryos were washed in PBS, fixed in 4% (v/v) paraformaldehyde in PBS for 15 min and mounted onto a glass microscope slide in a microdrop of Vectachield (Vector Laboratories, Inc., Peterborough, UK). Samples were examined with an Olympus Bx41 (Olympus, Hospitalet del Llobregat, Spain) epifluorescence microscope fitted with an image capture and analyzing system (Isis software version 5.4.5, Metasystems, Boston, USA). Images were acquired using the same exposure times and settings for all embryos and analyzed with ImageJ software (Image J 1.45q, Wayne Rasband, National Institutes of Health, Bethesda, USA) for fluorescence quantification. All individual nuclei were outlined for mean fluorescence intensity calculation.

### Embryo transfer

CD-1 female mice were mated with vasectomized males of the same strain and those with a vaginal plug were used as recipients at 0.5 day postcoitum. Between 9–10 two-cell cloned embryos were co-transferred with one parthenogenetic embryo into each oviduct of each female [48]. As control for the embryo transfer procedure, 6 *in vivo* fertilized embryos per oviduct were also transferred to other pseudo-pregnant recipients. The pups and their corresponding placentas were delivered by cesarian section at 19.5 days postcoitum and weighted in an analytical scale. Finally, the cloned pups were fostered to CD-1 mothers that had given birth the day before.

### Statistical analysis

Each experiment was repeated at least three times and the results obtained were pooled. Data on *in vitro* and *in vivo* embryonic development were analyzed by chi-square test or Fisher’s exact test. Comparisons of blastocyst cell numbers, fluorescence intensity and body and placenta weights were performed by Kruskall-Wallis test. In all cases, the GraphPad InStat program (version 3.05 for windows 95, GraphPad Software, La Jolla, USA) was used. A probability value of p < 0.05 was considered statistically significant.

## Results

### *In vitro* development and blastocyst quality of cloned embryos treated with VitC for various durations

In a first set of experiments, cloned embryos were treated with 100 μM VitC during 8–9 h, 16 h, 24 h and 120 h to determine the best treatment duration in terms of embryo development *in vitro* and blastocyst quality. In these experiments, CB was used during micromanipulation and activation procedures.

We found that the addition of VitC to embryo culture medium for at least 16 h post-activation significantly increased blastocyst rates and the mean number of ICM cells at 96 h post-activation compared with non-treated cloned embryos (Table 1). Blastocyst total cell numbers were also increased, but the results were only significant for the 16 h and 120 h treatments. Blastocyst formation rates and quality were not significantly affected by extending VitC treatment beyond 16 h, as 16 h, 24 h and 120 h treatments resulted in equivalent developmental rates and blastocyst cell numbers. Based on these results, we selected the shorter of these treatments (16 h) for further experiments.

**Table 1:** Effect of different durations of 100 μM VitC treatment on the *in vitro* development and quality of cloned mouse embryos.

			No. activated embryos developed to (%)				Mean cell number (±SEM) 96 h p.a.		
Group	No. reconstructed	No. activated (%)	Two cell	Morula	Blastocyst	No. blastocysts	ICM	TE	Total
NT	186	176 (94.6)^a^	143 (81.3)^a^ ^,^ ^b^	111 (63.1)^a^ ^,^ ^b^	47 (26.7)^a^	46	10.7±0.7^a^	24.9±1.5^a^	35.6±2.1^a^
VitC 8–9 h	206	200 (97.1)^a^	159 (79.5)^a^	112 (56.0)^a^	71 (35.5)^a^ ^,^ ^b^	39	12.8±0.9^a^ ^,^ ^b^	26.2±1.7^a^ ^,^ ^b^	39.0±2.4^a^ ^,^ ^b^
VitC 16 h	219	209 (95.4)^a^	188 (90.0)^c^	152 (72.7)^b^ ^,^ ^c^	89 (42.6)^b^ ^,^ ^c^	45	15.0±0.7^b^	31.5±1.3^a^ ^,^ ^b^	46.4±1.8^b^
VitC 24 h	218	208 (95.4)^a^	184 (88.5)^b^ ^,^ ^c^	149 (71.6)^b^ ^,^ ^c^	92 (44.2)^b^ ^,^ ^c^	43	14.3±0.7^b^	31.4±1.5^a^ ^,^ ^b^	45.7±2.1^a^ ^,^ ^b^
VitC 120 h	214	207 (96.7)^a^	186 (89.9)^c^	159 (76.8)^c^	95 (45.9)^c^	47	15.3±0.8^b^	32.8±1.5^b^	43.1±2.1^b^

NT: non-treated cloned embryos; p.a.: post-activation

^a-c^ Values with different superscripts differ significantly within the same column (p < 0.05; Chi-square test for embryonic development and Kruskall-Wallis for blastocysts cell numbers).

### *In vitro* development of cloned embryos treated with a combination of VitC and PsA

In a second set of experiments, aimed to investigate whether there is an additive effect between VitC and PsA treatments, cloned embryos were treated with 100 μM VitC, 10 μM PsA or the combination of both (VitC-PsA) during 16 h and their *in vitro* development was assessed. LatA, instead of CB, was used during micromanipulation and activation procedures in these experiments.

We found that the use of LatA significantly increased the blastocyst rate of non-treated cloned embryos (45.5%, Table 2) when compared with the use of CB in the previous experiments (26.7%, Table 1). In the case of VitC-treated embryos, blastocyst rates were also increased, but only moderately (52.1%, Table 2 *vs*. 42.6%, Table 1). As a result, no significant differences in blastocyst formation rates could be detected in this second set of experiments between non-treated and VitC-treated embryos. In spite of this, blastocyst rates in the non-treated group were significantly lower than those in the control ICSI group, whereas between the VitC-treated and the ICSI groups they were similar (Table 2).

**Table 2:** Effect of different epigenetic modifier treatments on the *in vitro* development of cloned mouse embryos.

			No. activated embryos developed to (%)		
Group	No. reconstructed	No. activated (%)	Two cell	Morula	Blastocyst
ICSI	252	248 (98.0)^a^	237 (95.6)^a^	170 (68.5)^a^ ^,^ ^b^	144 (58.1)^a^
NT	239	224 (93.7)^b^	181 (80.8)^b^	143 (63.8)^b^ ^,^ ^c^	102 (45.5)^b^ ^,^ ^c^
VitC 16 h	206	194 (94.2)^b^	174 (89.7)^c^	149 (76.8)^a^	101 (52.1)^a^ ^,^ ^b^
PsA 16 h	236	201 (85.2)^c^	166 (82.6)^b^ ^,^ ^c^	118 (58.7)^c^	83 (41.3)^c^
VitC-PsA 16 h	206	193 (93.7)^b^	164 (85.0)^b^ ^,^ ^c^	145 (75.1)^a^	109 (56.5)^a^

NT: non-treated cloned embryos

^a-c^ Values with different superscripts differ significantly within the same column (p < 0.05; Chi-square test)

The combined VitC-PsA treatment did not significantly improve the results obtained when using VitC alone, but it showed higher rates of blastocyst development than when using PsA alone (Table 2). Indeed, VitC resulted in significantly higher blastocyst rates than PsA. When compared to non-treated embryos, VitC-PsA was the only treatment in this second set of experiments producing higher blastocyst rates.

### Global histone acetylation and methylation levels and DNA methylation and hydroxymethylation levels in treated and non-treated cloned embryos

**Fig 1:**
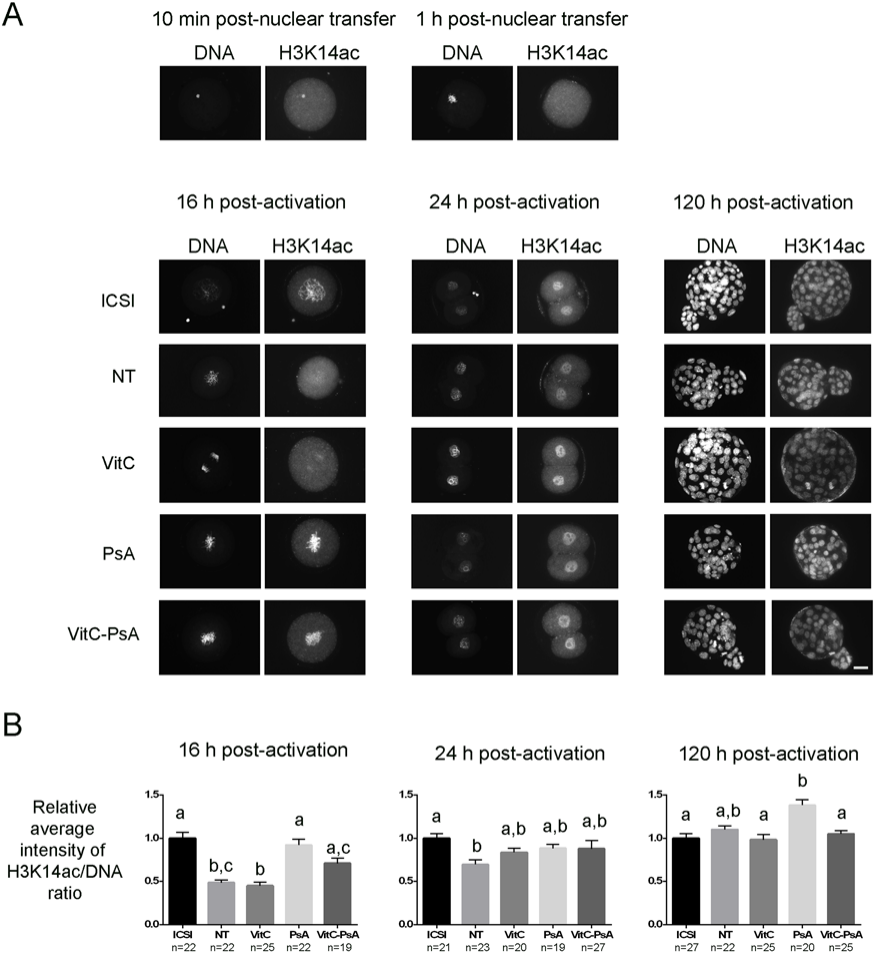
Psammaplin A, but not vitamin C, increases H3K14 acethylation at 16 h post-activation. ICSI embryos and cloned embryos non-treated (NT) and treated with 100 μM vitamin C (VitC), 10 μM psammaplin A (PsA) or the combination of both (VitC-PsA) during 16 h were immunostained for H3K14ac and DNA detection. A) Representative images of DNA and H3K14ac staining. Scale bar = 20 μm. B) Average intensity of H3K14ac/DNA signal ratio (+SEM) relative to ICSI embryos.

ICSI and cloned embryos were fixed at different developmental stages and immunostained for H3K14ac, H3K9me2, 5meC and 5hmeC marks. With regards to histone acetylation, we observed that H3K14 was highly acetylated in the cumulus cell nucleus 10 min after injection, but its acetylation markedly decreased 1 h after nuclear transfer into the enucleated oocyte (Fig. 1A). By the end of the treatment with the epigenetic modifiers (16 h post-activation), the VitC group displayed similar levels of H3K14ac as the non-treated group, both significantly lower than those of the ICSI group (Fig. 1). VitC-PsA-treated embryos were significantly more acetylated than embryos treated with VitC alone, at levels equivalent to those of PsA-treated cloned embryos and ICSI embryos. At the 2-cell stage (24 h post-activation), no significant differences were detected between treated and non-treated cloned embryos or among the three treated groups. In spite of this, only the three groups of treated embryos showed equivalent levels of H3K14ac to the control ICSI group. Finally, at the blastocyst stage (120 h post-activation), there were no differences between cloned and ICSI embryos, except for PsA-treated embryos which showed the highest levels of H3K14ac, even when compared with ICSI controls.

**Fig 2:**
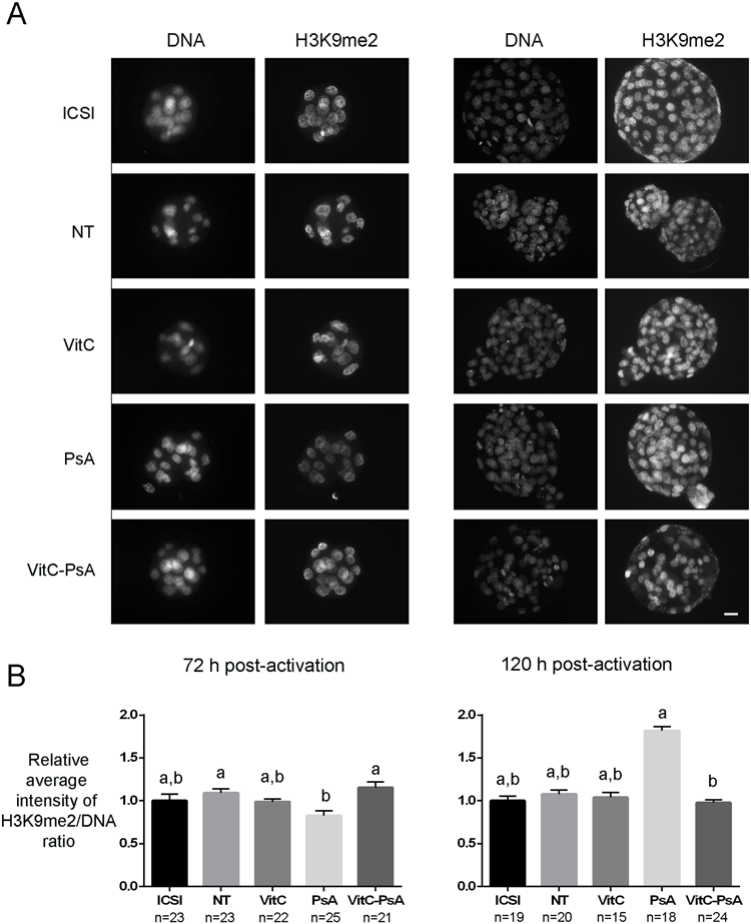
Psammaplin A, but not vitamin C, reduces H3K9 dimethylation at 72 h post-activation. ICSI embryos and cloned embryos non-treated (NT) and treated with 100 μM vitamin C (VitC), 10 μM psammaplin A (PsA) or the combination of both (VitC-PsA) during 16 h were immunostained for H3K9me2 and DNA detection. A) Representative images of DNA and H3K9me2 staining. Scale bar = 20 μm. B) Average intensity of H3K9me2/DNA signal ratio (+SEM) relative to ICSI embryos.

Levels of H3K9me2 were similar between ICSI and cloned embryos and between treated and non-treated cloned embryos at the two developmental stages analyzed, except for PsA-treated embryos which showed lower levels than the non-treated and the VitC-PsA groups by 72 h post-activation, and higher levels than the VitC-PsA group by 120 h post-activation (Fig. 2).

**Fig 3:**
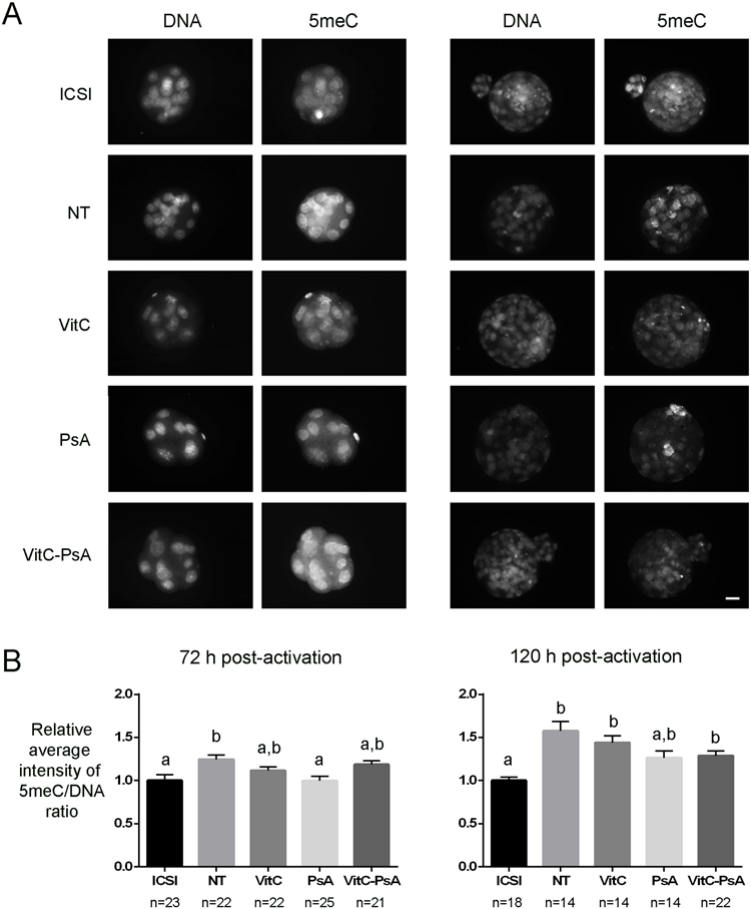
Psammaplin A, but not vitamin C, reduces DNA methylation at 72 h post-activation. ICSI embryos and cloned embryos non-treated (NT) and treated with 100 μM vitamin C (VitC), 10 μM psammaplin A (PsA) or the combination of both (VitC-PsA) during 16 h were immunostained for 5meC and DNA detection. A) Representative images of DNA and 5meC staining. Scale bar = 20 μm. B) Average intensity of 5meC/DNA signal ratio (+SEM) relative to ICSI embryos.

In the case of DNA methylation, we found that non-treated embryos were hypermethylated compared with ICSI embryos at both morula and blastocyst stages (Fig. 3). The three treatments showed a slight tendency to reduce methylation levels, although it was only statistically significant for PsA-treated morulae (72 h post-activation), which were significantly less methylated than non-treated ones. Whereas morulae from all three treatments had similar DNA methylation levels to ICSI ones, by the blastocyst stage (120 h post-activation) only the PsA-treated group maintained this similarity.

**Fig 4:**
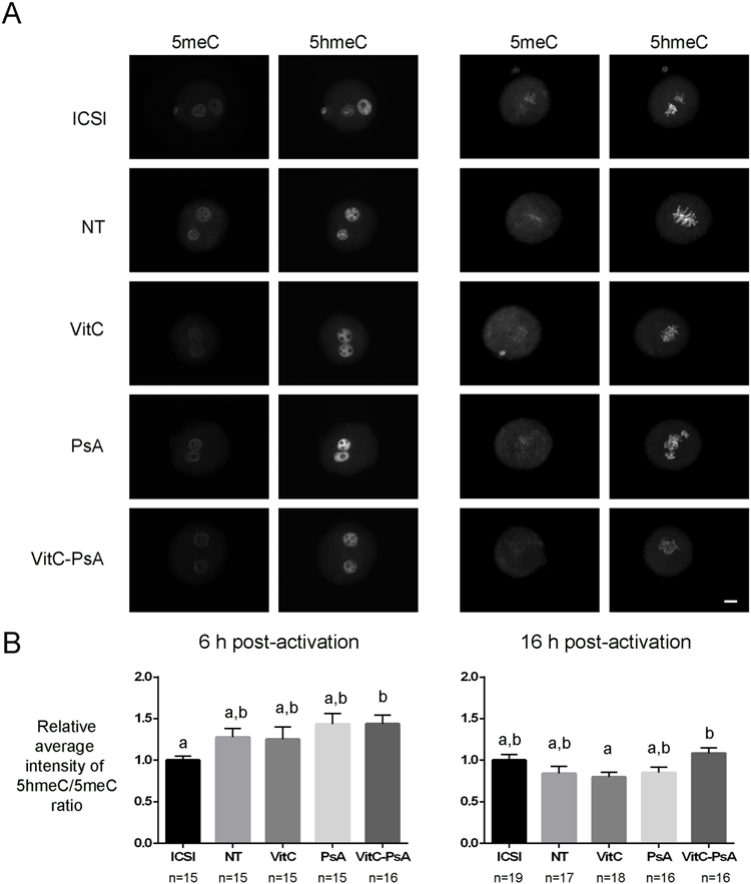
Vitamin C does not alter 5hmeC/5meC ratio. ICSI embryos and cloned embryos non-treated (NT) and treated with 100 μM vitamin C (VitC), 10 μM psammaplin A (PsA) or the combination of both (VitC-PsA) during 16 h were immunostained for 5meC and 5hmeC detection. A) Representative images of 5meC and 5hmeC staining. Scale bar = 20 μm. B) Average intensity of 5hmeC/5meC signal ratio (+SEM) relative to ICSI embryos.

Finally, we also analyzed DNA methylation and hydroxymethylation levels at previous stages of development, and we found that the ratio 5hmeC/5meC of cloned embryos was not affected by VitC or PsA treatments at 6 and 16 h post-activation (Fig. 4). However, a higher 5hmeC/5meC ratio was found in VitC-PsA treated embryos in comparison with VitC-treated ones at 16 h post activation.

### Levels of pluripotency and trophectoderm markers in treated and non-treated cloned embryos

In another set of experiments, ICSI and cloned embryos were fixed at 120 h post-activation and immunostained for OCT4 and NANOG pluripotency markers and the trophectoderm marker CDX2.

**Fig 5:**
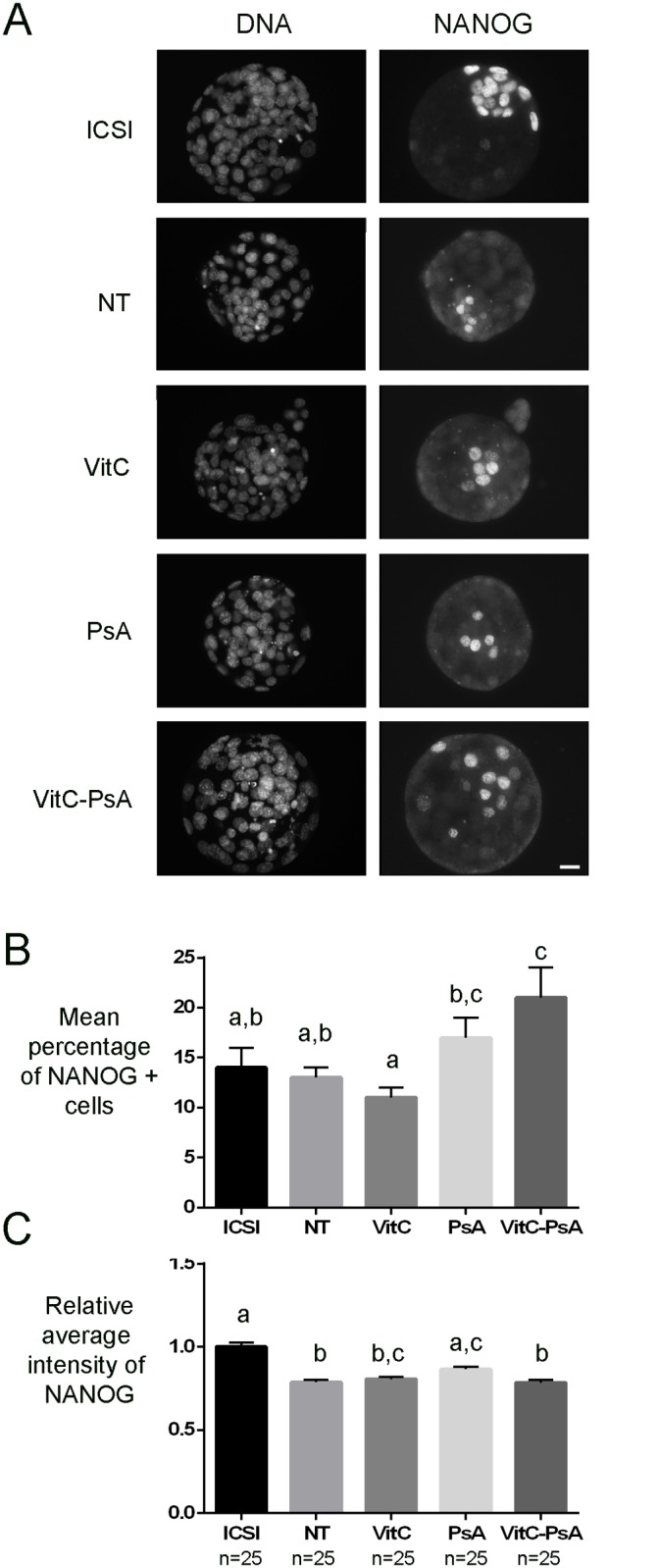
Psammaplin A, but not vitamin C, increases NANOG levels. ICSI blastocysts and cloned blastocysts non-treated (NT) and treated with 100 μM vitamin C (VitC), 10 μM psammaplin A (PsA) or the combination of both (VitC-PsA) during 16 h were immunostained for NANOG and DNA detection. A) Representative images of DNA and NANOG staining. Scale bar = 20 μm. B) Mean percentage of blastocyst cells (+SEM) positive for NANOG staining. C) Average intensity of NANOG signal (+SEM) relative to ICSI embryos.

**Fig 6:**
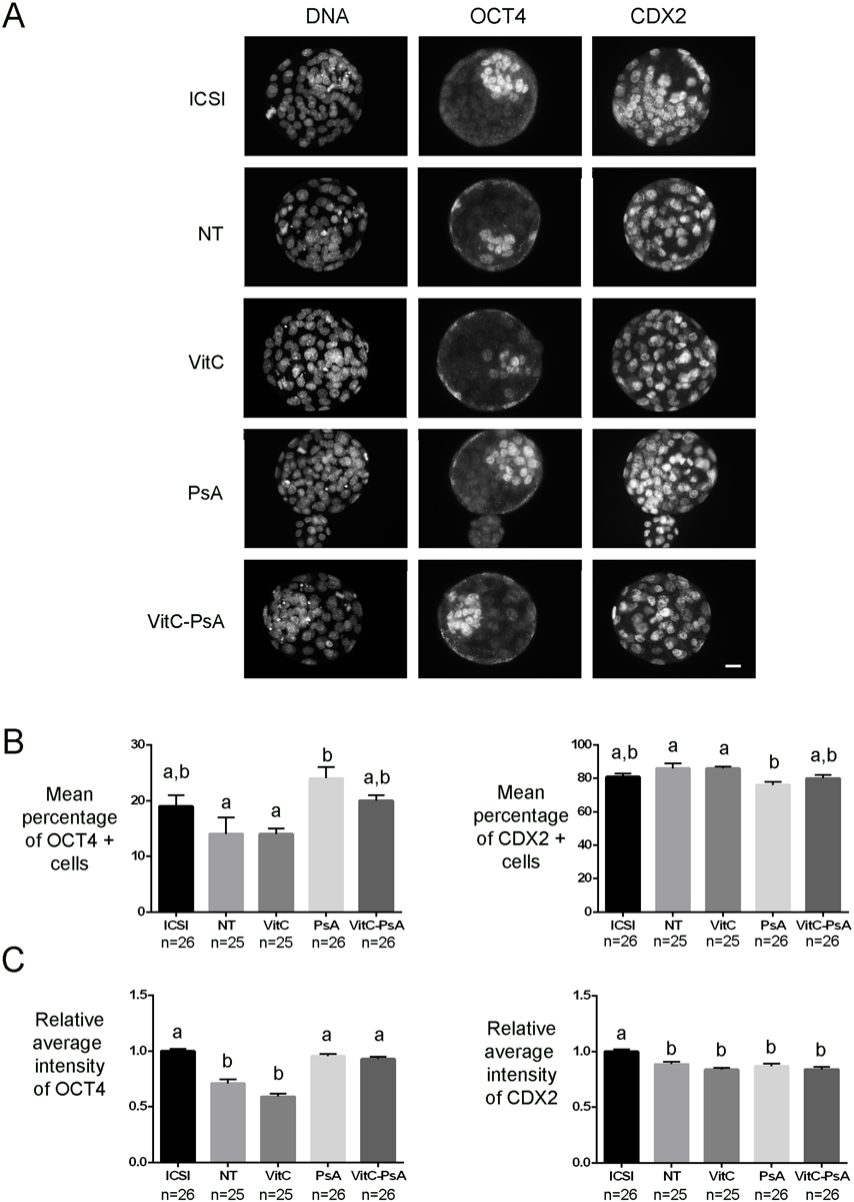
Psammaplin A alone or combined with vitamin C increases OCT4 but not CDX2 levels. ICSI blastocysts and cloned blastocysts non-treated (NT) and treated with 100 μM vitamin C (VitC), 10 μM psammaplin A (PsA) or the combination of both (VitC-PsA) during 16 h were immunostained for OCT4, CDX2 and DNA detection. A) Representative images of DNA, OCT4 and CDX2 staining. Scale bar = 20 μm. B) Mean percentage of blastocyst cells (+SEM) positive for OCT4 or CDX2 staining. C) Average intensity of OCT4 and CDX2 signals (+SEM) relative to ICSI embryos.

Although ICSI and non-treated cloned blastocysts had equivalent percentages of NANOG and OCT4 positive cells, global levels of NANOG and OCT4 were significantly lower in non-treated cloned blastocysts than in ICSI ones (Figs. 5 and 6). VitC treatment alone did not improve NANOG and OCT4 levels, nor the percentage of NANOG and OCT4 positive cells. In contrast, PsA treatment alone significantly increased the level of the two pluripotency markers, especially of OCT4 (1.3-fold increase), and the percentage of OCT4 positive cells, to levels equivalent to those of ICSI embryos. Cloned embryos undergoing the combined treatment VitC-PsA showed significantly increased levels of OCT4, but not of OCT4 positive cells, and a significantly higher percentage of NANOG positive cells, but not of NANOG levels.

With regards to CDX2, all groups of cloned embryos showed similar levels, which were significantly lower than in the ICSI group, and similar percentages of positive cells, which were equivalent to those of the ICSI group, except for PsA treated embryos which showed a lower percentage of CDX2 positive cells than non-treated and VitC-treated embryos (Fig. 6).

### Reduced glutathione content in treated and non-treated cloned embryos

**Fig 7:**
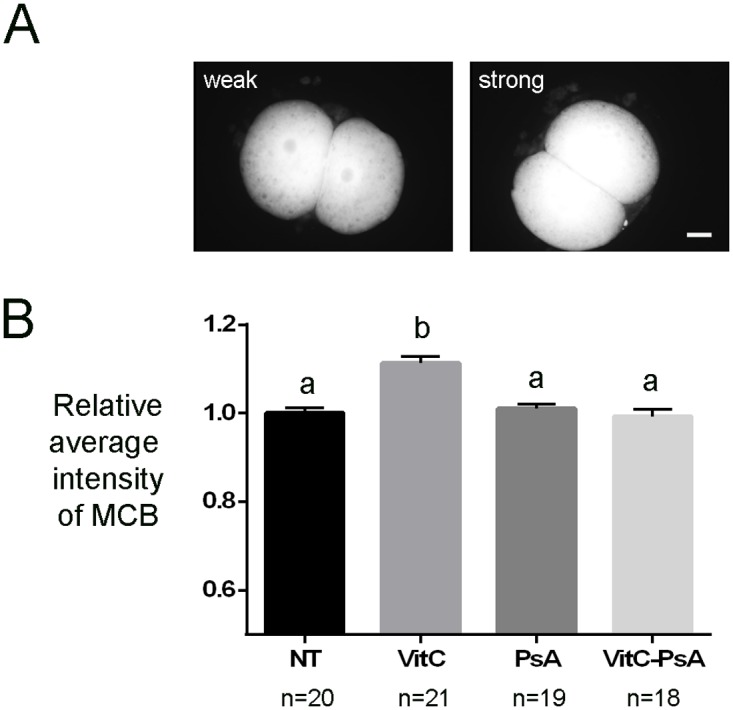
Vitamin C increases reduced glutathione content. Cloned embryos non-treated (NT) and treated with 100 μM vitamin C (VitC), 10 μM psammaplin A (PsA) or the combination of both (VitC-PsA) during 16 h were stained for reduced glutathione content detection at 16 h post-activation. A) Representative images of monochlorobimane (MCB) staining. Scale bar = 20 μm. B) Average intensity of MCB signal (+SEM) relative to non-treated embryos.

Cloned embryos were fixed at 16 h post-activation and stained for the quantification of GSH content as a measure of their reducing potential. We found that the levels of GSH were significantly higher in embryos treated with VitC alone than in all other groups of cloned embryos. In contrast, treatment with PsA did not alter the GSH content when compared with non-treated embryos (Fig. 7).

### Full-term development of treated and non-treated cloned embryos

Finally, in the last set of experiments cloned embryos were transferred, at the two-cell stage, to recipient females to assess their full-term development. We found that all three treatments resulted in significantly higher rates of full-term development when compared with the non-treated group, in which no live clones were born (Table 3). We also found a higher number of residual implantation sites in the recipient females from the treated groups. The number of pups produced was slightly higher in the VitC-PsA group, but not significantly different from the other two treatments. Irrespective of the treatment, both fetuses and placentas from the cloned groups were significantly heavier than those from the fertilized control group.

**Table 3:** Comparative full-term development and body and placenta weights of fertilized and cloned mouse embryos.

Group	No. 2-cell embryos transferred (recipients)	No. of live offspring (%)	No. of dead fetuses (%)	No. of residual implantation sites (%)	No. of placenta only (%)	Average weight (mg) (mean ± SD)	
						Body	Placenta
Control fertilized	36 (3)	23 (63.9)^a^	0 (0)^a^	5 (13.8)^a^	0 (0)^a^ ^,^ ^b^	1359.77 ± 20.12^a^	121.08 ± 4.78^a^
NT	258 (16)	0 (0)^b^	1 (0.4)^a^	9 (3.5)^b^	0 (0)^a^	-	-
VitC 16h	206 (13)	8 (3.9)^c^	0 (0)^a^	46 (22.3)^a^	4 (1.9)^b^	1667.99 ± 46.00^b^	298.71 ± 23.82^b^
PsA 16h	261 (17)	8 (3.1)^c^	1 (0.4)^a^	62 (23.75)^a^	0 (0)^a^	1561.84 ± 70.70^b^	299.78 ± 22.86^b^
VitC-PsA 16h	203 (13)	10 (4.9)^c^	0 (0)^a^	38 (18.7)^a^	1 (0.5)^a^ ^,^ ^b^	1618.34 ± 85.31^b^	298.54 ± 17.96^b^

NT: non-treated cloned embryos.

^a-c^ Values with different superscripts within the same column differ significantly (Fisher exact test for full-term development and Kruskall-Wallis test with Dunn post-test for body and placenta weights, p<0.05).

Of the 26 cloned pups produced, two apparently healthy females from the VitC treatment were cannibalized by the foster mother, one from the PsA treatment died shortly after birth due to respiratory problems, one from the VitC-PsA treatment was born prematurely by natural delivery and was found dead the next day, and three more females from the VitC-PsA treatment died prematurely at a few days of age due to unknown reasons. The other nineteen mice developed normally to adulthood and were able to reproduce when mated to normal B6CBAF1 males, giving rise to normal litters (3–12 pups).

## Discussion

In this study we were able to obtain the first mouse clones from SCNT embryos treated with VitC and with the combination VitC-PsA, using the actin polymerization inhibitor LatA during the micromanipulation and activation procedures. Along with PsA alone, all three treatments resulted in increased numbers of fertile cloned mice.

To determine the optimal duration of the VitC treatment for mouse SCNT, we chose a concentration of 100 μM, as it has been reported not to cause embryotoxicity when added to mouse embryo culture medium, whereas higher concentrations did [27, 41]. On the other hand, in spite that VitC concentrations over 200 μM have been shown to be beneficial for both *in vitro* and *in vivo* development of SCNT pig embryos in one study [40], Jeong et al. [29] found that only 100 μM VitC, but not lower (50 μM) or higher (200 μM) concentrations, improved blastocyst formation rates and blastocyst quality in porcine SCNT procedures. A VitC concentration of 227 μM has also been found to decrease the *in vitro* development rates of SCNT bovine embryos [42]. Among the different treatment durations tested in the present study, 16 h significantly increased blastocyst rates and ICM cell numbers and no further improvement was evident by extending the treatment. These results agree with the study of Huang et al. [40] in porcine SCNT embryos, in which 15 h of treatment with VitC after fusion was demonstrated to be enough for enhancing preimplantational development, but contrast with the study of Lee et al. [41] which reported that VitC does not increase the development of SCNT mouse embryos, probably due to the high concentration used.

Taking into account that reprogramming of the somatic nucleus is thought to occur mainly within a short period of time after nuclear transfer [49], whereas reactive oxygen species may accumulate during culture, the fact that a short treatment with VitC after parthenogenetic activation (16 h) is sufficient to improve cloned embryo development seems to reinforce the idea that the beneficial effects of VitC may be exerted through improvement of nuclear reprogramming rather than through its antioxidant activity [32]. Indeed, VitC has been reported to enhance JHDM and TET activities in mouse and human cells, promoting reduced levels of histone and DNA methylation [38]. In spite of this, we could not detect any clear improvement of VitC on the nuclear reprogramming markers H3K14ac, H3K9me2 and 5meC at the developmental stages analyzed, other than a slight increase in H3K14ac at 24 h post-activation and a slight decrease in DNA methylation at 72 h post-activation, to levels similar to those of ICSI embryos. Given that TET-mediated conversion of 5meC into 5hmeC may occur early in development [50], we also analyzed 5hmeC at earlier time-points after activation (6 and 16 h) but did not detect any effect of VitC on this marker either. Other authors have also failed to observe a substantial decrease in the global levels of 5meC in VitC-treated mouse embryonic stem cells (ESCs) [36] or cloned porcine embryos at 2-cell, 4-cell and blastocyst stages [51]. According to Blaschke et al. [36], TET-mediated VitC-induced demethylation seems to be restricted to specific genomic regions, whereas other regions such as repetitive elements, which cover a large portion of the genome, are resistant to VitC-induced DNA demethylation and can therefore obscure the results. In this sense, it is possible that the use of more sensitive and context-specific techniques of analysis than immunofluorescence would allow the detection of changes in DNA methylation levels in the treated embryos, as was indeed observed by Blaschke et al. [36] when performing 5meC immunoprecipitation followed by deep sequencing, or by Chung et al. [52] when using DNA methylation arrays in VitC-treated mouse and human ESCs, respectively. Alternatively, it is also possible that the effect of VitC on DNA methylation levels in preimplantational embryos is more limited than in ESCs, as Blaschke et al. [36] also reported that the stronger effect of VitC in mouse ESCs was found in regions that gain methylation in cultured ESCs compared to blastocysts, and that are methylated *in vivo* only after implantation. Similar arguments could explain the lack of effects of VitC on histone methylation levels observed in the present study, which contrasts with the reduced levels of H3K9 tri- and dimethylation reported after treatment of mouse pre-iPSCs with VitC [34]. With regards to histone acetylation, Huang et al. [40] reported increased levels of H4K5 acetylation in VitC-treated porcine SCNT embryos at the 2-cell, 4-cell and blastocyst stages. As VitC has not been shown to be a cofactor for acetyltransferases, and a direct effect of VitC on histone acetylation is therefore unlikely, histone acetylation levels could be indirectly altered through the effects of VitC on other epigenetic pathways, but no other epigenetic marks were analyzed in the study by Huang et al. [40]. In contrast, and in agreement with our results, Chawalit et al. [51] did not detect changes in H3K9 and K14 acetylation levels in handmade cloned porcine embryos continuously treated with VitC at any of the stages analyzed (2-cell, 4-cell and blastocysts). These differing results could be attributed to the specific lysines analyzed (H4K5 in Huang et al. [40] *vs* H3K9 and K14 in Chawalit et al. [51] and the present study), and deserve further investigation.

Consistent with the epigenetic results observed, the levels of pluripotency markers OCT4 and NANOG, which are often decreased in cloned embryos [53–55], were not improved in the blastocysts produced from the VitC-treated SCNT embryos. Again, our results are similar to those of Blaschke et al. [36] in VitC-treated mouse ESCs and of Chawalit et al. [51] in VitC-treated handmade cloned porcine embryos, but in disagreement with those of Huang et al. [40] demonstrating that OCT4 and SOX2 levels were elevated after treatment of cloned porcine embryos with VitC for 15 h after activation. It is noteworthy that despite the treatment with VitC apparently did not correct the abnormal levels of histone acetylation and of histone and DNA methylation, nor the defective expression of pluripotency genes, it resulted in a significant increase in full-term development of the treated SCNT embryos compared to the untreated ones. On the other hand, treatment of cloned embryos with the HDACi PsA also resulted in increased rates of full-term development, similar to those obtained with VitC, which in this case can be explained by its positive effects on nuclear reprogramming (increased levels of H3K14 acetylation, OCT4 and NANOG; decreased levels of DNA methylation). Nevertheless, none of the two epigenetic modifiers was able to correct placental overgrowth, a typical feature observed in cloned fetuses [56]. This could be related to an altered trophectoderm lineage, as also indicated by the low levels of CDX2, a transcription factor essential for trophectoderm and subsequent placenta formation [57], in all groups of cloned embryos, either treated or untreated.

It has been reported by Esteban et al. [32] that VitC and the HDACi valproic acid have an additive effect in improving nuclear reprogramming and mouse iPSCs generation. In contrast, no additive effect between VitC and another HDACi, trichostatin A, on nuclear reprogramming or blastocyst development was detected in handmade cloned porcine embryos [51]. Although we observed a slightly superior rate of full-term development when VitC and PsA treatments were combined than when using VitC or PsA separately, the additive effect of these two epigenetic modifiers on mouse SCNT was not evident. When compared to embryos treated with VitC alone, embryos treated with VitC-PsA showed some signs of improved nuclear reprogramming, such as increased levels of H3K14ac at 16 h post-activation and of OCT4 in the blastocysts produced, at levels equivalent to those of control ICSI embryos. This improvement can be attributed to the HDACi activity of PsA. Nonetheless, when compared to the treatment with PsA alone, some of the positive effects of PsA on nuclear reprogramming were not observed when the combined treatment was applied, even though *in vitro* development of the VitC-PsA treated embryos was improved. For instance, H3K14ac levels at 120 h post-activation were much lower in the VitC-PsA-treated embryos than in embryos treated with PsA alone, and the levels of NANOG, which were increased by the PsA treatment, were unaffected when VitC and PsA treatments were combined. Further investigation is required to elucidate the reasons for these unexpected results. On the other hand, the intracellular reduction of PsA is essential for its HDACi activity and it has been shown that GSH-depleted cells are not sensitive to PsA [44]. In this sense, we hypothesized that the antioxidant effect of VitC could increase embryo reducing potential, thus improving PsA reduction and, consequently, PsA-HDACi activity. However, in spite that VitC treatment increased GSH content in cloned embryos as expected from previous studies [30, 31, 51], no increase in histone acetylation levels or other synergistic effects with regards to PsA treatment were observed when VitC and PsA treatments were simultaneously applied. This suggests that the GSH content in the cloned embryos is not a limiting factor for the HDACi activity of PsA.

Another interesting finding of our study is the effect of LatA on the development of both treated and untreated cloned embryos. It has been previously reported that LatA increases full-term development of cloned mouse embryos when compared with CB [24, 25], as it corrects abnormal F-actin localization in cloned embryos and decreases the incidence of abnormal chromosome segregation [22, 24]. However, none of these previous studies analyzed the effects of LatA on *in vitro* development and in all of them the use of LatA was combined with the treatment of cloned embryos with an epigenetic modifier (either trichostatin A or PsA). To the best of our knowledge, this is the first report on the effects of LatA on the *in vitro* development of cloned embryos, both untreated and treated with an epigenetic modifier. Interestingly, we found that the use of LatA during micromanipulation and activation procedures increased blastocyst rates in the non-treated group, but not in the VitC-treated group, in which only a moderate increase was observed. A similar outcome is observed with PsA-treated embryos, in which blastocyst rates are similar with (41.3%, this study) or without (43.2%) [25] the use of LatA. In spite of their enhanced *in vitro* development, the full-term development of non-treated embryos was still suboptimal and no clones could be obtained. Therefore, our results seem to indicate that LatA enhances *in vitro* development in those groups of cloned embryos with a more impaired development (non-treated embryos), but this enhancement is not correlated with improved full-term development. It is possible that LatA corrects some abnormalities, such as chromosome segregation [22], with an impact on preimplantation development, but that other abnormalities, such as defective nuclear reprogramming, which are key for implantation and postimplantation development are not corrected by LatA. This would explain why LatA treatment is only effective in enhancing full-term rates when combined with an epigenetic modifier such as PsA, which has proved to improve nuclear reprogramming [this study and 25].

Cloning efficiencies obtained in the present study, both for non-treated and treated embryos, were not as high as those reported by other authors, which can reach up to 10% for HDACi-treated embryos. However, these reported data correspond to one of the mouse strains with higher cloning efficiencies (B6D2F1), and the effects of genotype on cloning rates are well-described [18]. Indeed, our null full-term developmental rates in the non-treated SCNT embryos agree with the results of other studies performed on the B6CBAF1 strain [14, 25, 58].

In summary, the present study demonstrates that: (1) VitC improves both *in vitro* and *in vivo* development of mouse SCNT embryos through a yet unknown mechanism, as no effects on nuclear reprogramming could be detected; (2) the combination of VitC with PsA does not show significant additive effects on mouse SCNT efficiency, although full-term development is slightly increased with regards to treatments with VitC or PsA alone; (3) the use of LatA during micromanipulation and activation procedures improves blastocyst formation in non-treated cloned embryos, but its enhancement of mouse cloning efficiencies requires the treatment of embryos with an epigenetic modifier.
